# Distribution of cup-disc ratio in a Swedish population

**DOI:** 10.48101/ujms.v128.9805

**Published:** 2023-10-13

**Authors:** Edvin Svedberg, Curt Ekström

**Affiliations:** Department of Surgical Sciences, Ophthalmology, Uppsala University, Uppsala, Sweden

**Keywords:** Epidemiology, heredity, intraocular pressure, open-angle glaucoma, optic disc, population survey, pseudoexfoliation, risk factor

## Abstract

**Background:**

Increased cup-disc ratio (CDR) is a hallmark of open-angle glaucoma (OAG), an age-related neurodegenerative disease of significant importance for public health. There are few studies on the distribution of CDR in the Nordic populations.

**Methods:**

The distribution of CDR was studied in 749 subjects aged 65–74 years in a population survey in the rural district of Tierp, Sweden, from 1984 to 86. The optic discs were assessed with binocular ophthalmoscopy at a slit lamp. Drawings of the discs were made in the protocol and used for the calculation of vertical CDRs. Odds ratios, adjusted for age and sex, according to Mantel-Haenszel (OR_MH_), were determined to estimate predictors of increased CDR, defined as a ratio in the upper quartile. For these analyses, the eye with the most advanced OAG or the highest pressure was chosen. Automated perimetry was used to identify OAG.

**Results:**

The distribution of vertical CDR was fairly close to that of other European-derived populations. The mean CDR was 0.45 in both eyes, with no difference between women and men. An increased ratio was associated with the age ≥70 years, a positive family history of OAG and intraocular pressure ≥20 mmHg. OAG increased the risk 8-fold (OR_MH_ 8.06; 95% CI 4.12–15.8).

**Conclusions:**

In this study, the distribution of CDR was fairly close to that of other European-derived populations. As expected, OAG increased the risk of having a CDR in the upper quartile. The CDR increased with age.

## Introduction

Open-angle glaucoma (OAG) is an age-related neurodegenerative disease of significant importance for public health, characterised by the progressive loss of optic nerve fibres with cupping of the optic disc and consistent visual field defects. Glaucoma is a leading cause of irreversible blindness worldwide (1). In Sweden, increased intraocular pressure (IOP) and pseudoexfoliation (PEX) are important risk factors for the development of OAG (2). In PEX, a fibrillar material is produced and accumulated in the anterior eye segment (3). Common sequence variants in the lysyl oxidase-like 1 gene, involved in elastic fibre formation, are closely related to PEX (4).

Increased cup-disc ratio (CDR) is a hallmark of OAG and has been suggested to be a part of the classification of OAG in population surveys (5). The CDR, defined as the diameter of the optic cup divided by the diameter of the optic disc, is usually measured vertically. In most population surveys, the CDR has been assessed by photographic methods. In clinical practice, optical coherence tomography or confocal scanning laser ophthalmoscopy are advised for diagnostic purposes (6). However, if an imaging technique is not available, a detailed manual drawing of the discs has been recommended (7).

The size of the normal optic disc is subject to substantial variation. Furthermore, the CDR is strongly related to the size of the disc (8). Larger discs have larger cups than smaller discs. Consequently, a large CDR in a large normal disc is easily mistaken as glaucomatous, whilst a small CDR in a small glaucomatous disc may be classified as normal, as demonstrated in a Swedish study (9).

The distribution of CDR is well-known from numerous studies on different ethnicities. Results from four studies on European-derived populations are presented in Table 1 (10–13). All studies reported a mode of 0.3–0.5. To the best of our knowledge, the Reykjavik Eye Study was the only study of its kind done in a Nordic country. Notably, PEX was a common finding in Reykjavik but not in the other studies.

**Table 1:** Distribution of vertical cup-disc ratio in the right eyes in four population studies.

Study	Method	Mode	Median	Mean
Beaver Dam^a^	Photography	0.3	0.36	–
Blue Mountains^b^	Photography	0.4	–	0.43
Melbourne^c^	Slit lamp examination	0.3	–	0.38
Reykjavik^d^	Photography	0.5	–	–

Method: Method used to assess the cup-disc ratio.

a Ref. (10);

b Ref. (11);

c Ref. (12);

d Ref. (13).

The objectives of the present research were to examine the distribution of CDR in a Swedish population with a high exposure to PEX and to estimate predictors of an increased CDR. The investigation took the form of a cross-sectional study on a defined population.

## Methods

### The Tierp Glaucoma Survey

From 1984 to 1986, a population survey was conducted in the rural district of Tierp, south central Sweden. Its target population comprised 2,429 residents, aged 65-74-years-old. A sample of about one-third of the target population was randomly selected. Of the eligible number of 838 individuals, 760 (90.7%) underwent a detailed eye examination, as described elsewhere (14). Briefly, an interview was first held, covering medical and family history. The pressure was taken with a Goldmann applanation tonometer mounted on a Haag–Streit slit lamp. As a rule, the pressure was taken with single tonometer readings. If the difference between the two eyes exceeded 2 mm Hg, a control measurement was done. In this case, the second reading was defined as the IOP for that person. The visual fields were examined using the Competer 350 automated perimeter (Bara Elektronik AB, Lund, Sweden). After perimetry, the pupils were dilated, and slit lamp biomicroscopy, including a binocular assessment of the optic discs and gonioscopy, was done. PEX was defined as the presence of characteristic white flakes on the lens capsule or on the pupillary border.

### The study population

**Figure 1:**
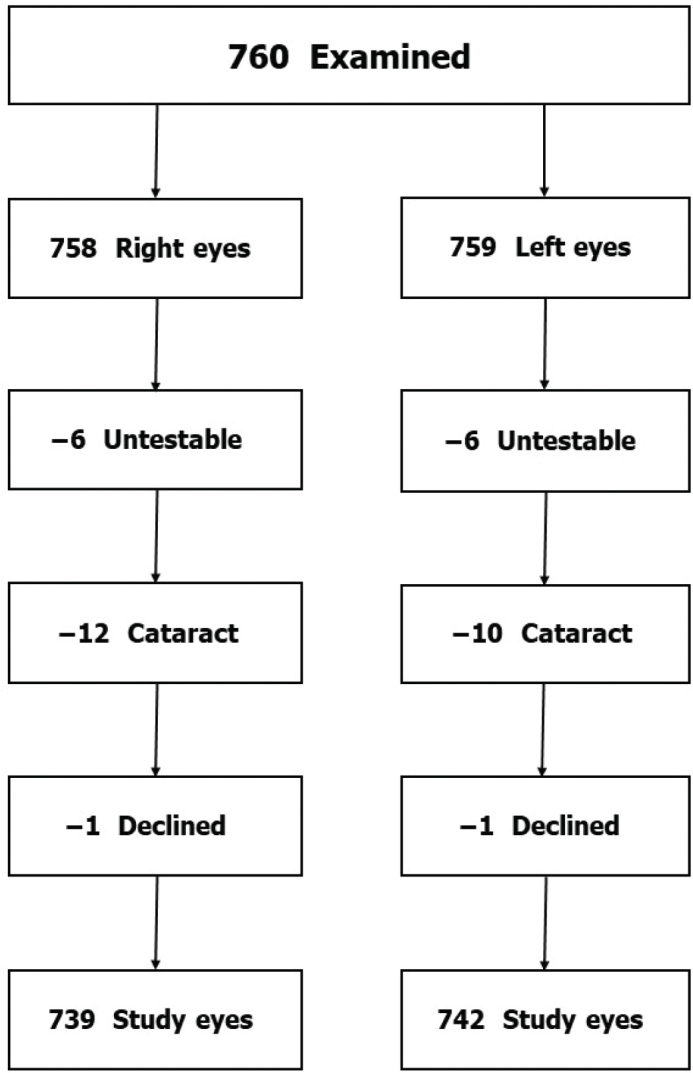
Flow chart showing how the study samples of 739 right eyes and 742 left eyes in the Tierp Glaucoma Survey were derived.

Of the total number of 760 participants, the right eye had been removed in two subjects and the left eye in one subject, leaving 758 right and 759 left eyes for analyses. Six people were impossible to examine, as were the optic discs of 12 right and 10 left eyes with pronounced cataract. The eyes of these subjects were excluded from the analyses of the respective eye. One subject declined the instillation of eye drops (Figure 1). The remaining 739 right eyes and 742 left eyes were used for the calculation of CDRs.

For the analyses of predictors of an increased CDR, the eye with the most advanced OAG, or with the highest pressure, designated as ‘the eye under study’, was chosen. One patient with angle-closure glaucoma and two patients with secondary glaucoma in either eye were excluded, and one individual had dense cataract in both eyes. As mentioned earlier, six people were impossible to examine, and one declined. Thus, 749 individuals provided information for this part of the study. The investigation was approved by the Ethics Committee at the Faculty of Medicine, Uppsala University, on 11 May 1983 and adhered to the tenets of the Declaration of Helsinki. An informed consent was obtained from all participants.

### Assessment of CDR

**Figure 2:**
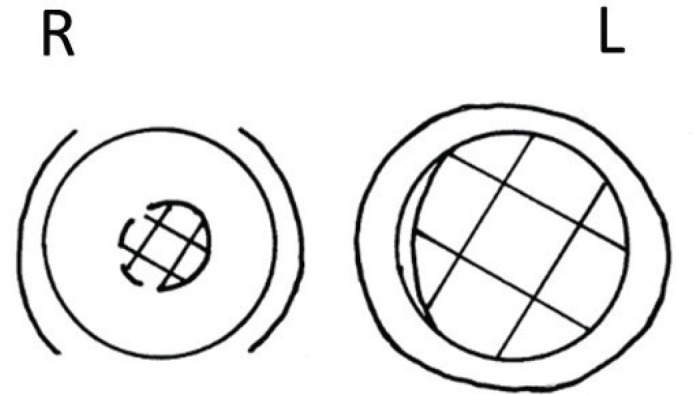
Drawing of the optic discs in a patient with open-angle glaucoma in the left eye. Parapapillary findings are indicated. R = right eye; L = left eye.

The optic discs were examined with binocular ophthalmoscopy at the slit lamp using a Goldmann single mirror contact lens. Drawings of the optic discs were made in the protocol according to a modification of a method suggested by Shaffer et al. (15). The drawings were made on the basis of shape rather than colour. The disc margin was defined as the inner margin of the scleral ring, and the diameter of the cup was estimated from the point at which the disc surface made its first definite transition posteriorly (Figure 2). The CDR was calculated by dividing the optic cup diameter by the optic disc diameter. The size of the optic disc in the protocol was 15.5 mm. The cup diameter was obtained by measuring the maximal extent of the cup through the centre of the disc between 45–135° and 225–315° by the authors in a joint session, using a ruler to the accuracy of 1 mm. Finally, the vertical CDR was calculated by dividing the cup diameter by 15.5.

### Classification of OAG

Consistent with the concept of Foster et al. (5), glaucoma with PEX was classified as OAG. To qualify for a diagnosis of OAG, a reproducible visual field defect was a prerequisite, consistent with glaucoma and not explicable on other grounds, as described elsewhere (14).

### Assessment of systemic predictors

Information on treated systemic hypertension, ischaemic heart disease and diabetes mellitus was obtained at the interview or from medical records. In the case of a discrepancy between the self-reported history and the medical record, data from the latter source were used in this report. The participants were asked if they were current smokers or past smokers and when they stopped smoking. Information on smoking was also acquired from medical records and family members.

### Statistical methods

The agreement between repeated CDR measurements was evaluated using kappa statistics. Thus, the readings of the first 100 right and left eyes in the sample were repeated, and kappa coefficients for a cup diameter ≥8 mm were calculated. Predictors of an increased CDR, defined as a ratio in the upper quartile, were estimated using 2 × 2 tables, with odds ratios adjusted for age and sex strata, according to the Mantel–Hansel’s method (OR_MH_). To simultaneously assess several variables affecting the risk of having an increased CDR, multiple logistic regression analyses were used, with a CDR in the upper quartile as the dependent variable.

## Results

**Figure 3:**
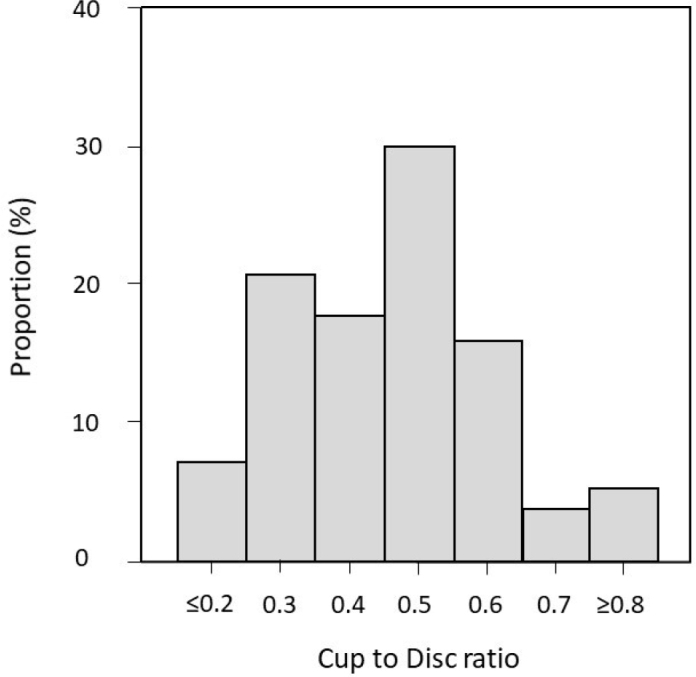
Distribution of the vertical cup to disc ratio in 749 eyes in the Tierp Glaucoma Survey. The eye with the most advanced open-angle glaucoma, or the highest intraocular pressure, was chosen. The optic disc in both eyes of 11 people was not examined.

The mean vertical CDR was 0.45 in the right and left eyes (Table 2). There was a tendency for increasing CDRs with age. No difference was observed between women and men. The median was 0.45 in both eyes, with a ratio of 0.52 enclosing the upper quartile. The distribution of CDRs in the eye under study is shown in Figure 3. The measurements were approximately normally distributed. The kappa coefficients were 0.98 and 0.93 for the right and left eyes, respectively.

**Table 2:** Distribution of mean vertical cup-disc ratios in the right and left eyes in the Tierp Glaucoma Survey by age and sex.

Age	Females		Males		All	
	Right	Left	Right	Left	Right	Left
	(*n* = 382)	(*n* = 385)	(*n* = 357)	(*n* = 357)	(*n* = 739)	(*n* = 742)
65–69 years	0.42	0.42	0.43	0.43	0.43	0.42
70–74 years	0.48	0.47	0.47	0.48	0.47	0.48
65–74 years	0.45	0.44	0.45	0.46	0.45	0.45

Forty-three subjects fulfilled a diagnosis of OAG. PEX in either eye was present in 105 subjects (14.0%), of whom 15 had OAG. Odds ratios for a CDR in the upper quartile are presented in Table 3. Substantial risks were observed for age ≥70 years, a positive family history of OAG, increased IOP and OAG. A pressure of 20–24 mmHg increased the risk of having a CDR in the upper quartile 1.6-fold, whilst a pressure ≥25 mmHg increased the risk 4-fold. A ratio in the upper quartile was associated with an 8-fold increased risk of having glaucoma. No significant effects were found for male sex, PEX, smoking habits, diabetes, hypertension or ischaemic heart disease. The result of a logistic regression model including age, family history of OAG, IOP and ischaemic heart disease was close to that of the stratified analyses (data not shown).

**Table 3:** Odds ratios for cup-disc ratios in the upper quartile in the eye under study in the Tierp Glaucoma Survey, adjusted for age and sex.

Characteristics	No. of cases			
		(*n* = 185)	OR_MH_	(95% CI)
Age ≥70 years^a^	No	82	1.00	
	Yes	103	1.62	(1.16–2.27)
Male sex^b^	No	95	1.00	
	Yes	90	1.03	(0.74–1.44)
Family history, open-angle glaucoma^b^	No	168	1.00	
	Yes	17	2.24	(1.18–4.24)
Intraocular pressure, mmHg^b^	<20	132	1.00	
	20–24	39	1.60	(1.03–2.49)
	≥25	14	4.06	(1.85–8.92)
Open-angle glaucoma	No	155	1.00	
	Yes	30	8.06	(4.12–15.8)
Pseudoexfoliation, either eye	No	156	1.00	
	Yes	29	1.21	(0.76–1.93)
Smoking status	Never smoked	125	1.00	
	Past smoker	41	1.17	(0.74–1.87)
	Current smoker	19	0.55	(0.32–0.95)
Diabetes mellitus	No	159	1.00	
	Yes	26	1.20	(0.74–1.97)
Hypertension, treated	No	137	1.00	
	Yes	48	0.86	(0.59–1.26)
Ischaemic heart disease	No	148	1.00	
	Yes	37	1.54	(0.99–2.38)

CI: confidence interval; OR_MH_: Mantel–Haenszel adjusted odds ratio.

a Adjusted for sex;

b adjusted for age.

The eye under study includes the eye with the most advanced open-angle glaucoma or the highest pressure.

## Discussion

To the best of our knowledge, only one population-based study has previously been reported on the distribution of CDR in the Nordic countries, namely, the Reykjavik Eye Study (13), whilst the Tierp study is the second. The study in Reykjavik used stereo photographs for grading of the optic discs, whilst the study in Tierp utilised drawings made under binocular ophthalmoscopy at the slit lamp. Despite the different methods, the distribution of CDR was fairly close to that of other European-derived populations using photographic methods. For this reason, we believe that the method used in Tierp was an appropriate alternative to more costly methods. Furthermore, we are not aware of other studies reporting the use of drawings for measuring the CDR.

Clearly, the methods are important for obtaining results comparable with other studies. Thus, for people aged 65–74 years, the Framingham Eye Study (16) reported a mean vertical CDR of 0.25 in the eyes screened, compared with a mean of 0.45 in the present study. Most likely, the different methods for assessing the CDR account for the inconsistent results, as the disc was viewed using indirect ophthalmoscopy with a 14 dioptre lens in the Framingham study. In accordance with the majority of studies referred to in the present report, the distribution of CDR approximately followed a normal distribution. Nevertheless, there was an evident dip in the graph of CDRs with a measure of 0.4. Possibly, the limited sample size was a reasonable explanation for this finding.

In this study, increased IOP and OAG were strongly related to a CDR in the upper quartile. Similar results have been reported in other population studies (17). Considering the well-known relationship between increased IOP and the risk of developing OAG (2), this finding was not surprising. Furthermore, we confirmed an already known association between age and increased CDR. In a population survey from southern Sweden, Bengtsson observed a marked increase in the cup diameter with ageing (18). The disc diameter increased slowly with advancing age, but the rim area remained unaffected. Another study on healthy volunteers reported an increase in the mean vertical CDR of about 0.1 between the ages of 30 and 70 years (19). In the present study, however, subjects aged ≥70 years were examined about 1 year earlier than those aged <70 years. It cannot be ruled out that the judgement of the discs had changed whilst the study was in progress, and in some way affected the results. A family history of OAG was associated with a two-fold increased risk of having a CDR in the upper quartile. The recognised relationship between a positive family history and OAG (20, 21) is a likely explanation for this finding.

Our study has several strengths, including its population-based design, high participation rate and the use of a detailed protocol. All eye examinations at the slit lamp, including the drawing of the optic discs, were conducted by the same glaucoma specialist, who was masked to the result of the pressure readings and the visual field testing. The kappa statistics indicated an excellent agreement between repeated CDR measurements. Furthermore, a visual field defect was required for a diagnosis of OAG. Nevertheless, as with many epidemiologic studies, the research was limited in several respects.

Most importantly, compared with many other population studies, the Tierp Glaucoma Survey was a small study, limiting its statistical power to provide reliable estimates on some of the predictors of increased CDR. However, the drawing of optic discs gave sufficient data for an accurate description of the distribution of CDRs in the examined population, which was the main issue of this study. Although more sophisticated methods are available at present, the results obtained in Tierp were fairly close to that of other studies using photos for grading of the disc. Moreover, this study only involved people aged 65–74-years-old, which might be a limitation with respect to the reported increase in CDR with age.

There is a risk of misclassification of exposure in cross-sectional studies when data are based on self-reports, which was the case regarding smoking habits. This type of information bias should be non-differential, thereby ‘diluting’ the relationship between increased CDR and possible predictors in the analyses. Besides, as mentioned earlier, participants aged ≥70 years were examined about 1 year earlier than the rest of the sample, with the possibility of observational bias in the judgement of the optic discs. On the other hand, the fact that the drawings were performed by a single ophthalmologist might be a strength of this study, as it eliminates the risk of inter-observer variation.

In conclusion, in this population-based study on individuals aged 65–74-years-old in Sweden, the distribution of vertical CDR was fairly close to that of other studies on European-derived populations. As expected, increased IOP and OAG were strongly correlated to a CDR in the upper quartile. The CDR increased with age.

## References

[1] Tham YC, Li X, Wong TY, Quigley HA, Aung T, Cheng CY. Global prevalence of glaucoma and projections of glaucoma burden through 2040: a systematic review and meta-analysis. Ophthalmology. 2014;121(11): 2081–90. doi: 10.1016/j.ophtha.2014.05.013 24974815

[2] Ekström C. Risk factors for incident open-angle glaucoma: a population-based 20-year follow-up study. Acta Ophthalmol. 2012;90(4): 316–21. doi: 10.1111/j.1755-3768.2010.01943.x 20626719

[3] Ritch R, Schlötzer-Schrehardt U. Exfoliation syndrome. Surv Ophthalmol. 2001;45(4):265–315. doi: 10.1016/s0039-6257(00)00196-x 11166342

[4] Thorleifsson G, Magnusson KP, Sulem P, Walters GB, Gudbjartsson DF, Stefansson H, et al. Common sequence variants in the LOXL1 gene confer susceptibility to exfoliation glaucoma. Science. 2007;317(5843): 1397–400. doi: 10.1126/science.1146554 17690259

[5] Foster PJ, Buhrmann R, Quigley HA, Johnson GJ. The definition and classification of glaucoma in prevalence surveys. Br J Ophthalmol. 2002;86(2):238–42. doi: 10.1136/bjo.86.2.238 11815354 PMC1771026

[6] Quigley HA. Glaucoma. Lancet. 2011;377(9774):1367–77. doi: 10.1016/S0140-6736(10)61423-7 21453963

[7] European Glaucoma Society. Terminology and guidelines for glaucoma. 5th ed. Savona; PubliComm; 2021, p. 77. 10.1136/bjophthalmol-2021-egsguidelines 34675001

[8] Bengtsson B. The variation and covariation of cup and disc diameters. Acta Ophthalmol (Copenh). 1976;54(12):804–18. doi: 10.1111/j.1755-3768.1976.tb01801.x 990030

[9] Heijl A, Mölder H. Optic disc diameter influences the ability to detect glaucomatous disc damage. Acta Ophthalmol. 1993;71(1):122–9. doi: 10.1111/j.1755-3768.1993.tb04974.x 8475706

[10] Klein BEK, Klein R, Sponsel WE, Franke T, Cantor LB, Martone J, et al. Prevalence of glaucoma. The Beaver Dam Eye Study. Ophthalmology. 1992;99(10):1499–504. doi: 10.1016/s0161-6420(92)31774-9 1454314

[11] Mitchell P, Smith W, Attebo K, Healey PR. Prevalence of open-angle glaucoma in Australia. The Blue Mountains Eye Study. Ophthalmology. 1996;103(10):1661–9. doi: 10.1016/s0161-6420(96)30449-1 8874440

[12] Wensor MD, McCarty CA, Stanislavsky YL, Livingston PM, Taylor HR. The prevalence of glaucoma in the Melbourne Visual Impairment Project. Ophthalmology. 1998;105(4):733–9. doi: 10.1016/S0161-6420(98)94031-3 9544649

[13] Jonasson F, Damji KF, Arnarsson A, Sverrisson T, Wang L, Sasaki H, et al. Prevalence of open-angle glaucoma in Iceland: Reykjavik Eye Study. Eye. 2003;17(6):747–53. doi: 10.1038/sj.eye.6700374 12928689

[14] Ekström C. Prevalence of open-angle glaucoma in central Sweden. The Tierp Glaucoma Survey. Acta Ophthalmol Scand. 1996;74(2):107–12. doi: 10.1111/j.1600-0420.1996.tb00052.x 8739672

[15] Shaffer RN, Ridgway WL, Brown R, Kramer SG. The use of diagrams to record changes in glaucomatous disks. Am J Ophthalmol. 1975;80(3):460–4. doi: 10.1016/0002-9394(75)90534-6 1163592

[16] Leibowitz HM, Krueger DE, Maunder LR, Milton RC, Kini MM, Kahn HA, et al. The Framingham Eye Study monograph: an ophthalmological and epidemiological study of cataract, glaucoma, diabetic retinopathy, macular degeneration, and visual acuity in a general population of 2631 adults, 1973–1975. Surv Ophthalmol. 1980;24(Suppl):335–610. doi: 10.1016/0039-6257(80)90122-8 7444756

[17] Pakravan M, Yazdani S, Javadi M-A, Amini H, Behroozi Z, Ziaei H, et al. A population-based survey of the prevalence and types of glaucoma in central Iran: the Yazd eye study. Ophthalmology. 2013;120(10):1977–84. doi: 10.1016/j.ophtha.2013.02.029 23664464

[18] Bengtsson B. The alteration and asymmetry of cup and disc diameters. Acta Ophthalmol. 1980;58(5):726–32. doi: 10.1111/j.1755-3768.1980.tb06685.x 7211261

[19] Garway-Heath DF, Wollstein G, Hitchings RA. Aging changes of the optic nerve head in relation to open angle glaucoma. Br J Ophthalmol. 1997;81(10):840–5. doi: 10.1136/bjo.81.10.840 9486023 PMC1722014

[20] Wolfs RC, Klaver CC, Ramrattan RS, van Duijn CM, Hofman A, de Jong PT. Genetic risk of primary open-angle glaucoma: population-based familial aggregation study. Arch Ophthalmol. 1998;116:1640–5. doi: 10.1001/archopht.116.12.1640 9869795

[21] Jonas JB, Aung T, Bourne RR, Bron AM, Ritch R, Panda-Jonas S. Glaucoma. Lancet. 2017:390(10108):2183–93. doi: 10.1016/S0140-6736(17)31469-1 28577860

